# Associations of Inosine with Gut Microbiota, Metabolic Indicators, and Fluid Homeostasis in Kidney-Related Diarrhea

**DOI:** 10.3390/ijms27156540

**Published:** 2026-07-23

**Authors:** Huiyi Peng, Qin Liu, Zhoujin Tan

**Affiliations:** 1 School of Traditional Chinese Medicine, Hunan University of Chinese Medicine, Changsha 410208, China; penghy25@163.com (H.P.); liuqin18874743502@163.com (Q.L.); 2 Hunan Key Laboratory of Traditional Chinese Medicine Prescription and Syndromes Translational Medicine, Changsha 410208, China

**Keywords:** inosine, gut microbiota, metabolism, inflammation, diarrhea

## Abstract

As a natural purine metabolite, inosine’s impact on kidney-related diarrhea through its influence on gut microbiota and associated metabolic functions remains unclear. Kidney-related diarrhea was induced in male KM mice. Histopathological alterations and inflammatory infiltration were assessed using hematoxylin and eosin (HE) staining. ELISA was used to measure corticosterone (CORT), antidiuretic hormone (ADH), and adenosine triphosphate (ATP) to assess metabolic status and fluid homeostasis. Immunohistochemistry (IHC) staining techniques, Real-Time Quantitative Polymerase Chain Reaction (RT-qPCR), and Western blot (WB) were used to analyze the expression of aquaporin-4 (AQP4), AMP-activated protein kinase (AMPK), nuclear factor kappa-B (NF-κB), and adenosine A2A Receptor (A2AR). Gut microbiota composition and predicted functional pathways were analyzed using 16S rRNA sequencing and KEGG-based functional prediction, followed by correlation analyses between the microbiota and factors. Inosine improved renal function, alleviated renal and colonic histopathological damage, and reduced inflammatory infiltration. It also increased ATP, ADH, and CORT levels, indicating improvements in metabolic and fluid-balance-related factors. Inosine also increased A2AR, AMPK, and AQP4 expression while decreasing NF-κB expression. Moreover, inosine altered gut microbial composition and was associated with differences in predicted microbial functional pathways. Significant correlations were observed between specific bacterial taxa and host indicators. Inosine alleviated kidney-related diarrhea in mice, accompanied by improvements in metabolic status and fluid homeostasis, reduced inflammation, modulation of A2AR/AMPK/NF-κB-related signaling pathway, and alterations in gut microbial composition. Further studies are required to clarify the causal contributions of gut microbial composition and functional activity to the beneficial effects of inosine.

## 1. Introduction

Inosine is a naturally occurring purine nucleoside produced by adenosine deamination and acts as a bioactive metabolite in purine metabolism [1]. It is mainly obtained from nucleic acid-rich foods, such as meat and edible fungi [2,3], and can also be generated via microbiota-associated purine metabolism [4]. Inosine can be converted to inosine monophosphate (IMP), thereby contributing to adenosine monophosphate (AMP) synthesis and adenosine triphosphate (ATP) regeneration [1,5]. Among microbiota-associated metabolites, inosine has attracted considerable attention for its involvement in anti-inflammatory, antioxidant, immunomodulatory, and metabolic regulatory activities [6,7,8].

The anti-inflammatory properties of inosine are closely associated with the activation of the adenosine A2A Receptor (A2AR) [8]. As a G protein-coupled receptor extensively distributed in tissues including the kidneys and intestines, A2AR suppresses inflammatory responses via a cAMP-dependent signaling pathway [9]. Moreover, A2AR can activate AMP-activated protein kinase (AMPK) under certain conditions [10]. AMPK, a key molecule in cellular energy sensing and inflammation regulation, upon activation, inhibits the nuclear factor kappa-B (NF-κB) signaling pathway, which in turn leads to a decrease in the release of pro-inflammatory cytokines like tumor necrosis factor alpha (TNF-α) [11,12]. Therefore, A2AR/AMPK/NF-κB signaling pathway may contribute to the anti-inflammatory and metabolic regulatory effects of inosine and may participate in the maintenance of metabolic homeostasis.

The gut microbiota is a complex microbial ecosystem that plays a critical role in host health. Gastrointestinal dysfunctions, including diarrhea, are highly prevalent among patients with kidney diseases and are frequently associated with heightened inflammatory responses, metabolic disturbances, and alterations in gut microbiota composition [13,14,15]. To investigate the pathophysiological mechanisms underlying kidney-related diarrhea, our group previously established a mouse model induced by adenine and *Folium sennae*. This model reproduces the major pathological features of kidney-related diarrhea, including persistent diarrhea, renal and intestinal injury, inflammation, and gut microbiota dysbiosis. Therefore, this model is suitable for investigating the interactions between gut microbiota, metabolic homeostasis, and kidney-related diarrhea. Using this model, we previously demonstrated close associations among energy metabolism, immune regulation, and inflammatory responses, highlighting the potential contribution of the gut microbiota to the pathogenesis of kidney-related diarrhea [16,17,18]. Microbiota-derived metabolites can link microbial activity with host energy metabolism, immunity, and endocrine regulation [19,20]. Current evidence indicates that exogenous inosine administration can ameliorate colonic inflammation in ulcerative colitis and modulate gut microbial composition by modulating the NF-κB signaling pathway [21,22]. However, the association between inosine, gut microbiota, and metabolic homeostasis during kidney-related diarrhea remains unclear.

In the present study, we used the adenine- and *Folium sennae*-induced mouse model to investigate the therapeutic effects and underlying mechanisms of inosine in kidney-related diarrhea. We hypothesized that inosine alleviates kidney-related diarrhea by remodeling the gut microbiota, restoring metabolic homeostasis, and regulating the A2AR/AMPK/NF-κB signaling pathway. To test this hypothesis, we assessed inflammatory responses and metabolic and fluid balance parameters in the colon, kidney, and serum; simultaneously, we utilized 16S rRNA sequencing and bioinformatics to analyze the gut microbiota composition, microbiota-associated metabolic pathways, and their correlation with host metabolic markers.

## 2. Results

### 2.1. General Condition of Each Group of Mice

**Figure 1:**
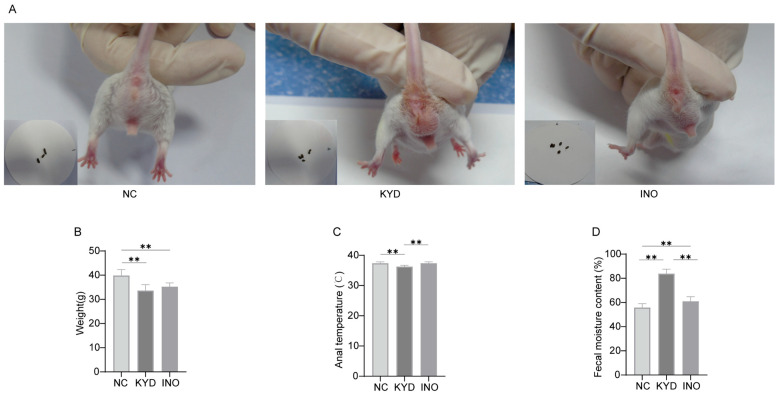
(**A**) Anal cleanliness and fecal characteristics of mice. (**B**) Body weight, (**C**) rectal temperature, and (**D**) fecal moisture content of mice (n = 10 per group). ** *p* < 0.01.

Mice in the normal control (NC) group showed normal activity and responsiveness, and clean perianal regions. In contrast, kidney-related diarrhea model (KYD) mice exhibited reduced activity and responsiveness, loose stools, and perianal fecal contamination. These abnormalities were partially alleviated by inosine treatment, which improved stool characteristics and reduced perianal contamination (Figure 1A).

As shown in Figure 1B, body weight significantly decreased following adenine and Folium sennae administration (*p* < 0.01). Inosine treatment partially restored body weight, although values remained lower than those of the NC group (*p* < 0.01). Rectal temperature was significantly lower in the KYD group than in the NC and inosine intervention (INO) groups (*p* < 0.01) (Figure 1C). Similarly, fecal moisture content was highest in the KYD group, intermediate in the INO group, and lowest in the NC group (*p* < 0.01) (Figure 1D).

### 2.2. The Impact of Inosine on Structural and Functional Alterations in the Kidney

**Figure 2:**
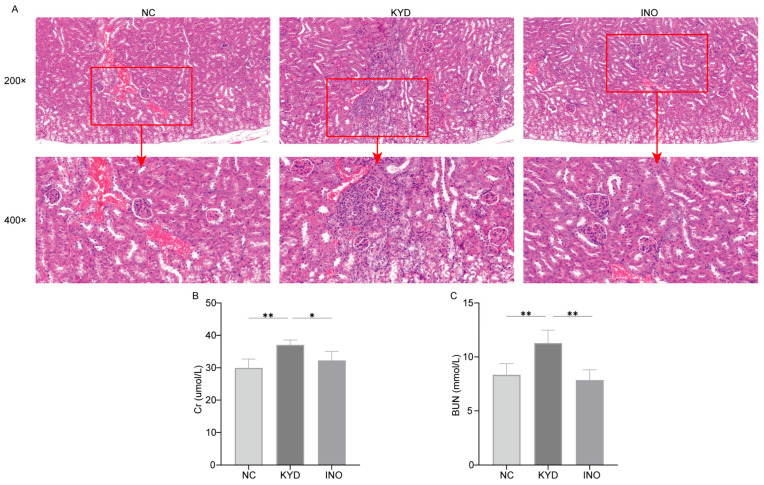
Kidney histology and function. (**A**) Kidney sections stained with hematoxylin and eosin (HE) (200×, 400×). (**B**) Serum Cr and (**C**) BUN levels (n = 5 per group). * *p* < 0.05, ** *p* < 0.01.

As shown in Figure 2A, the kidney structure of the mice in the NC group was clear and complete, with no marked inflammatory cell infiltration or structural changes. In contrast, after the modeling was completed, the mice in the KYD group exhibited renal tubular dilation, cellular edema, and the aggregation of cellular debris within the tubules. Moreover, there was significant inflammatory cell infiltration in the glomeruli and interstitium. In the INO group of mice treated with inosine, although renal tubular dilation and cellular edema were still observed, there was no obvious inflammatory cell infiltration or structural damage. Additionally, the levels of creatinine (Cr) and blood urea nitrogen (BUN) in the KYD group were significantly elevated (*p* < 0.01), and compared to the KYD group, the levels of Cr and BUN in the INO group mice were significantly decreased (*p* < 0.05, *p* < 0.01). Therefore, inosine may have ameliorated the kidney damage.

### 2.3. The Impact of Inosine on Structural Changes in the Colon

**Figure 3:**
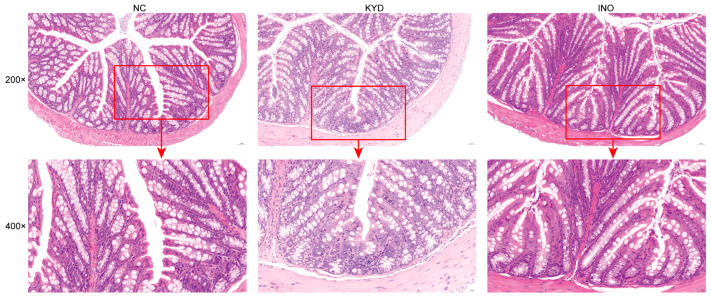
Colon structure of mice after HE staining (200×, 400×).

The colonic structures of the three groups of mice are shown in Figure 3. In the NC group, the colon tissue and cellular morphology remained intact, with no evidence of inflammatory cell infiltration. Conversely, the KYD group displayed inflammatory cell infiltration within the colon tissue, and the structure of the gut gland cells was indistinct. In the INO group, however, the colon tissue structure was preserved, showing no signs of tissue damage or significant inflammatory cell infiltration. These findings suggest that gavage with adenine and *Folium sennae* can damage colon tissue cells in mice, and that histopathological damage was reduced in the INO group.

### 2.4. The Impact of Inosine on CORT, ADH, and ATP Levels in Mice

**Figure 4:**
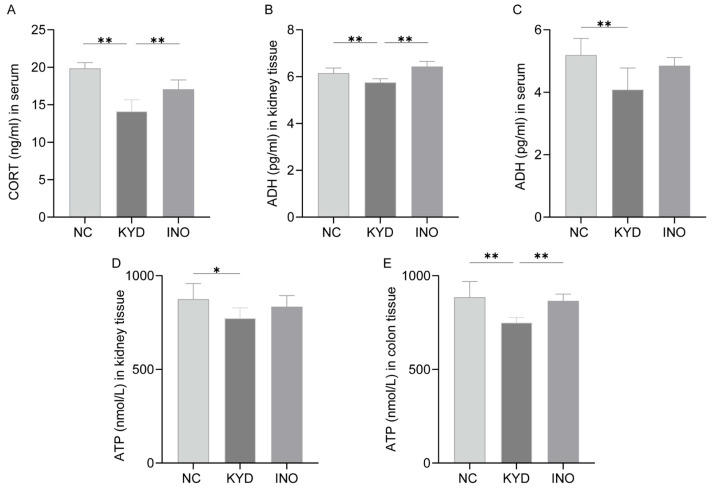
(**A**) serum CORT levels, (**B**) kidney tissue ADH levels, (**C**) serum ADH levels, (**D**) kidney tissue ATP levels, and (**E**) colon tissue ATP levels in each group of mice (n = 6 per group). * *p* < 0.05, ** *p* < 0.01.

As shown in Figure 4A, serum corticosterone (CORT) levels were significantly reduced in the KYD group compared with the NC and INO groups (*p* < 0.01), whereas no significant difference was observed between the NC and INO groups (*p* > 0.05). Similar results were observed for renal antidiuretic hormone (ADH) levels (Figure 4B), with the KYD group exhibiting significantly lower levels than both the NC and INO groups (*p* < 0.01). Serum ADH levels (Figure 4C) were highest in the NC group and lowest in the KYD group, with a significant difference between these groups (*p* < 0.01), while the increase in the INO group did not reach statistical significance (*p* > 0.05). ATP levels in colon tissue (Figure 4D) were significantly reduced in the KYD group compared with both the NC and INO groups (*p* < 0.01). In kidney tissue (Figure 4E), ATP levels were significantly lower in the KYD group than in the NC group (*p* < 0.01), whereas the increase following inosine treatment was not statistically significant (*p* > 0.05).

### 2.5. The Effect of Inosine on AMPK and NF-κB mRNA Levels in Colon and Kidney

**Figure 5:**
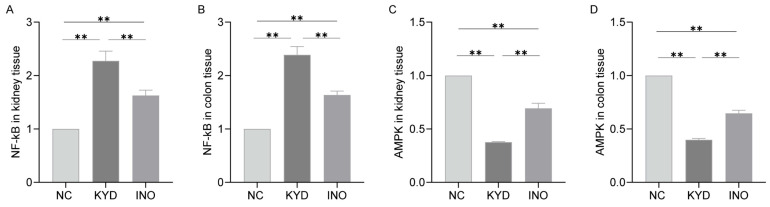
RT-qPCR (n = 3 per group). NF-κB expression levels in (**A**) the kidney tissues and (**B**) the colon tissues of mice in each group. AMPK expression levels in (**C**) the kidney tissues and (**D**) the colon tissues of mice in each group. ** *p* < 0.01.

The mRNA expression of NF-κB in the KYD group was markedly higher than that in the other two groups (*p* < 0.01), whereas the NC group exhibited significantly lower NF-κB mRNA levels compared to the others (*p* < 0.01). In addition, AMPK mRNA expression was substantially higher in the NC group than in the KYD and INO groups (*p* < 0.01), with the KYD group showing the lowest expression level, followed by the INO group (*p* < 0.01). These findings demonstrate that adenine and *Folium sennae* increased NF-κB transcription while decreasing AMPK transcription in the colonic and renal tissues of the mice. Notably, this effect was partially reversed by inosine intervention (Figure 5).

### 2.6. The Effect of Inosine on A2AR Protein Expression Levels in Colon and Kidney

**Figure 6:**
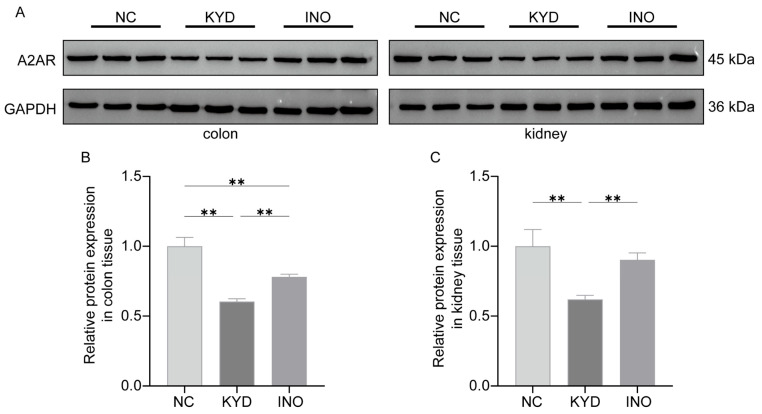
WB analysis (n = 3 per group). (**A**) The expression levels of A2AR in the colon and kidney. Relative protein expression of A2AR in (**B**) colon tissues and (**C**) kidney tissues of mice in each group. ** *p* < 0.01.

Significant differences in A2AR expression were found in the colon and kidney tissues among the various groups of mice (Figure 6). Compared with the INO and NC groups, the KYD group exhibited lower relative A2AR protein expression in both colon and kidney tissues (*p* < 0.01). Following inosine intervention in the INO group, A2AR protein expression in colon tissue was elevated compared with the KYD group, though it remained below that of the NC group (*p* < 0.01). In contrast, the A2AR level in the kidney tissue of the INO group did not differ significantly from that of the NC group (*p* > 0.05). The ability of inosine to upregulate A2AR expression indicates that it can counteract the suppression of A2AR in the colon and kidneys induced by the combined adenine and *Folium sennae* gavage.

### 2.7. The Effect of Inosine on AQP4 Expression in the Colon and Kidney

**Figure 7:**
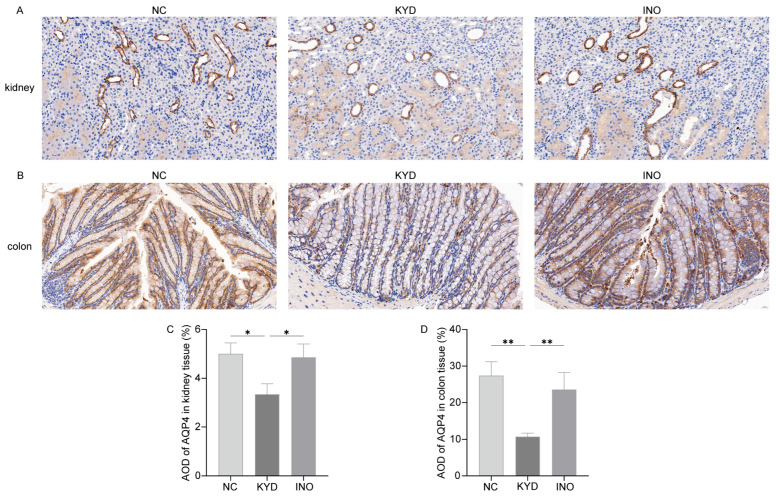
IHC of AQP4 expression in (**A**) colon and (**B**) kidney tissue (n = 3 per group, 400×). Average optical density (AOD) value of AQP4 in (**C**) kidney and (**D**) colon tissue. * *p* < 0.05, ** *p* < 0.01.

As illustrated in Figure 7, AQP4 expression was highest in the kidney and colon tissues of the NC group and lowest in those of the KYD group, with the difference being statistically significant (*p* < 0.05, *p* < 0.01). Relative to the KYD group, the INO group exhibited an increase in AQP4 expression in both the colon and kidney tissues (*p* < 0.05, *p* < 0.01). While the AQP4 expression level in the INO group was marginally lower than that in the NC group, this difference did not reach statistical significance (*p* > 0.05).

### 2.8. The Effect of Inosine on the Gut Microbiota in Mice

#### 2.8.1. Gut Microbial Diversity in Each Group of Mice

**Figure 8:**
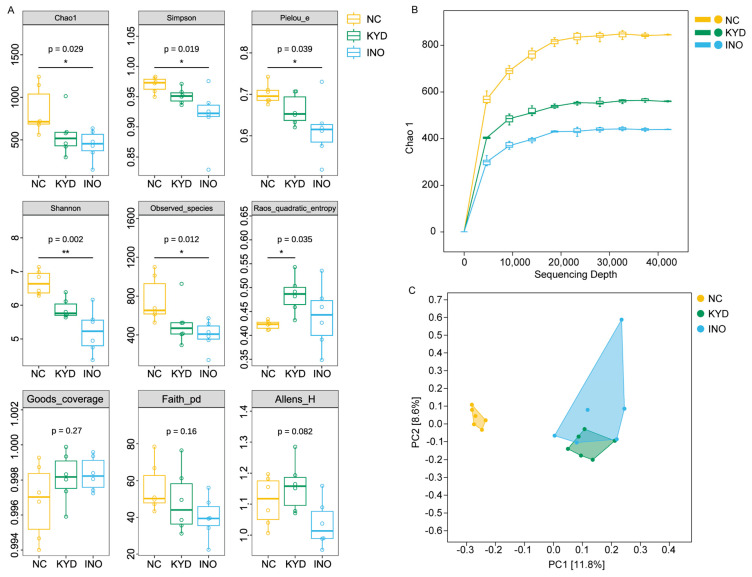
Diversity of Gut Microbiota (n = 6 per group). (**A**) Alpha diversity. (**B**) Rarefaction curve. (**C**) Beta diversity analyzed by PCoA. * *p* < 0.05, ** *p* < 0.01.

Both Chao1 and Observed species indices (Figure 8A) showed that gut microbial richness was highest in NC, lower in KYD, and lowest in INO, with a significant difference between NC and INO (*p* < 0.05), but not between NC and KYD (*p* > 0.05). Shannon and Simpson indices followed the same order, with a significant difference between NC and INO (*p* < 0.05), while KYD and INO were not significantly different (*p* > 0.05). Roa’s quadratic entropy index was significantly higher in KYD than NC (*p* < 0.05); INO showed a decrease relative to KYD but an increase relative to NC, without statistical significance (*p* > 0.05). Pielou’s evenness index was highest in NC, followed by KYD and INO, with a significant difference between NC and INO (*p* < 0.05), while KYD vs. NC was not significant (*p* > 0.05). These results indicate that NC mice had the richest and most even gut microbiota, KYD mice had greater variation, and INO intervention partially restored richness and evenness toward NC levels.

As shown in the Rarefaction curve in Figure 8B, under identical sequencing depths, the NC group exhibits the highest alpha diversity, while the INO group shows the lowest. The curve plateaus at a sequencing depth of 40,000, signifying that this depth is adequate, and that the alpha diversity analysis has captured nearly all species. Beta diversity analysis via principal coordinate analysis (PCoA) revealed that the NC group is significantly separated from both the KYD and INO groups. In contrast, the KYD and INO groups cluster closely together with overlapping sample points, indicating no significant structural difference between them. This demonstrates that the microbiota community structure in the INO group did not return to a state resembling that of the NC group and did not become structurally distinct from the KYD group.

#### 2.8.2. Composition and Differences in the Gut Microbiota of Mice

**Figure 9:**
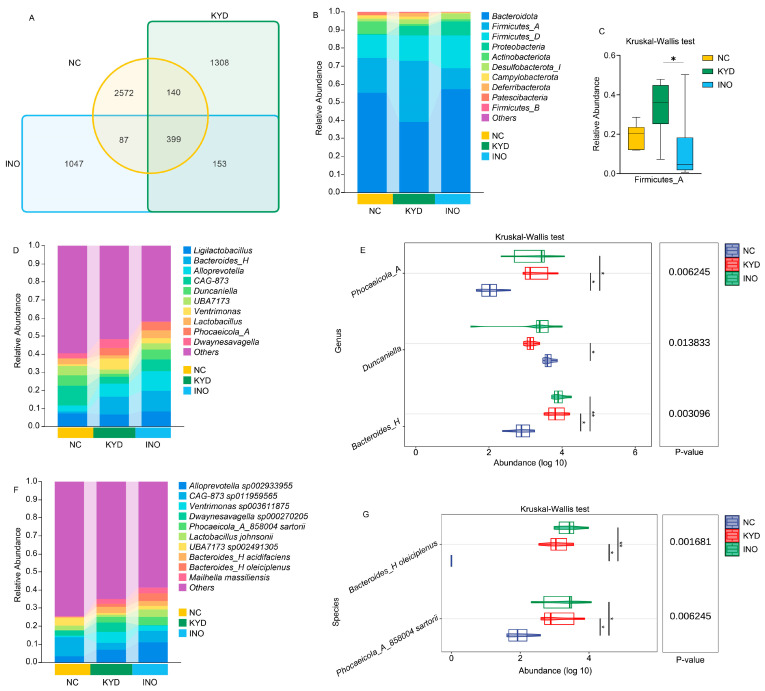
Different levels of composition and differences in gut microbiota (n = 6 per group). (**A**) Venn diagram. (**B**) Top 10 phyla. (**C**) Inter-group compositional differences at the phylum level. (**D**) Top 10 genera. (**E**) Inter-group compositional differences at the genus level. (**F**) Top 10 species. (**G**) Inter-group compositional differences at the species level. * *p* < 0.05, ** *p* < 0.01.

The gut microbial composition, as measured by Amplicon Sequence Variants (ASVs) abundance, was compared across the three mouse groups (Figure 9A). The NC, KYD, and INO groups had total ASV counts of 2572, 1308, and 1047, respectively. A total of 399 ASVs were shared across all three groups. The NC group shared 140 ASVs with the KYD group and 87 ASVs with the INO group, while the KYD and INO groups shared 153 ASVs. Subsequently, the top 10 microbial communities at each taxonomic level were selected for each group to analyze their composition and differences.

At the phylum level (Figure 9B), the top 10 phyla were: Bacteroidota, Firmicutes_A, Firmicutes_D, Proteobacteria, Actinobacteriota, Desulfobacterota_I, Campylobacterota, Deferribacterota, Patescibacteria, and Firmicutes_B. Bacteroidota and Firmicutes_A showed notable numerical variations across the three groups. The relative abundances of Bacteroidota in the NC, KYD, and INO groups were 55.21%, 39.09%, and 57.20%, respectively. However, these differences did not reach statistical significance. Meanwhile, the relative abundances of Firmicutes_A in the NC, KYD, and INO groups were 19.26%, 33.75%, and 11.71%, respectively. The relative abundance of Firmicutes_A was significantly lower in the INO group than in the KYD group (*p* < 0.05) (Figure 9C). These results indicate that inosine significantly reduced the abundance of Firmicutes_A compared with the KYD group, while the changes in Bacteroidota represented only a numerical trend.

At the genus level (Figure 9D), the top 10 genera were *Ligilactobacillus*, *Bacteroides_H*, *Alloprevotella*, *CAG-873*, *Duncaniella*, *UBA7173*, *Ventrimonas*, *Lactobacillus*, *Phocaeicola_A*, and *Dwaynesavagella. Duncaniella* abundance was highest in NC and lowest in KYD, with a significant difference between NC and KYD (*p* < 0.05), while INO showed a non-significant recovery. This finding suggests that the abundance of *Duncaniella* was significantly reduced in the diarrhea model. *Phocaeicola_A* and *Bacteroides_H* were significantly lower in NC than in KYD and INO (*p* < 0.05, *p* < 0.01). To further investigate, we profiled the top ten bacterial species (Figure 9F) and compared them across groups (Figure 9G). Among these species, both *Phocaeicola_A_858004 sartorii* and *Bacteroides_H oleiciplenus* exhibited a consistent pattern: their levels were lowest in the NC group, significantly higher in the KYD and INO groups (*p* < 0.05, *p* < 0.01), though no significant difference was found between the KYD and INO groups (*p* > 0.05).

#### 2.8.3. The Effect of Inosine on the Characteristic Gut Microbiota

**Figure 10:**
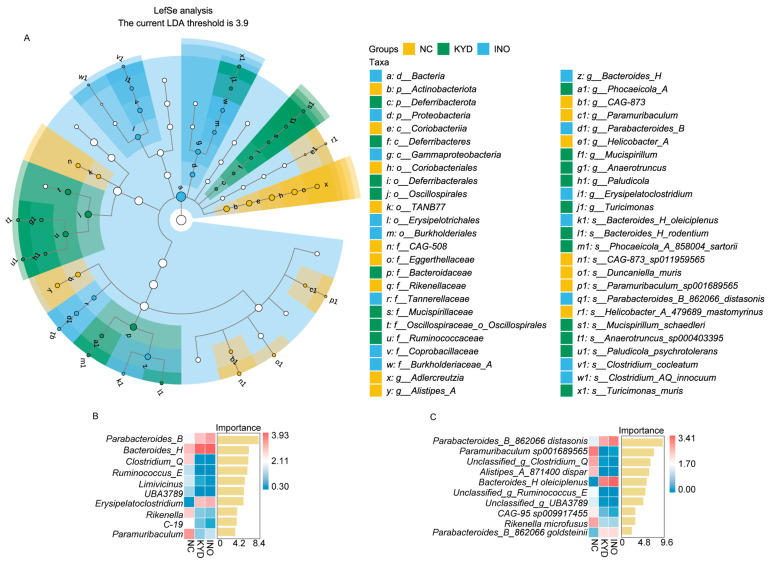
Characteristic gut microbiota (n = 6 per group). (**A**) Cladogram from LefSe analysis. Random forest plots at (**B**) the genus level and (**C**) species level.

We employed linear discriminant analysis effect size (LefSe) analysis to assess differential features across all taxonomic levels for all samples within the three groups (Figure 10A). The LDA score was set to a minimum threshold of 2, with the final threshold set to 3.9, indicating a substantial effect size for the observed differences. At the phylum level, the phyla that significantly differentiated the NC, KYD, and INO groups were Actinobacteriota, Deferribacterota, and Proteobacteria, respectively. At the genus level, the species showing significant differences in the NC group include *CAG-873*, *Paramuribaculum*, *Alistipes_A*, *Helicobacter_A*, and *Adlercreutzia*. In the KYD group, the significantly different bacterial genera were *Phocaeicola_A*, *Mucispirillum*, *Turicimonas*, *Anaerotruncus*, and *Paludicola.* For the INO group, the genera with significant differences were *Bacteroides_H*, *Parabacteroides_B*, and *Erysipelatoclostridium.* Furthermore, at the species level, significant differences were observed in the NC group for *CAG-873 sp011959565*, *Paramuribaculum_sp001689565*, *Helicobacter_A_479689_mastomyrinus*, and *Duncaniella muris*. In the KYD group, the significantly different species were *Mucispirillum_schaedleri*, *Turicimonas_muris, Phocaeicola_A_858004 sartorii, Anaerotruncus_sp000403395, Bacteroides_H_rodentium*, and *Paludicola_psychrotolerans.* Finally, for the INO group, the species with significant differences were *Bacteroides_H_oleiciplenus*, *Parabacteroides_B_862066_distasonis*, *Clostridium_cocleatum*, and *Clostridium_AQ_innocuum*.

Subsequently, we performed a random forest analysis to assess the importance of genera and species within each group. The yellow bar charts in the right panel display the importance scores for each species (Figure 10B,C). The analysis revealed that the top 10 most significant genera, ranked from highest to lowest, were *Parabacteroides_B*, *Bacteroides_H*, *Clostridium_Q*, *Ruminococcus_E*, *Limivicinus*, *UBA3789*, *Erysipelatoclostridium*, *Rikenella*, *C-19*, and *Paramuribaculum*. The corresponding top species were *Parabacteroides_B_862066 distasonis*, *Paramuribaculum sp001689565, Unclassified_g_Clostridium_Q*, *Alistipes_A_871400 dispar*, *Bacteroides_H oleiciplenus*, *Unclassified_g_Ruminococcus_E*, *Unclassified_g_UBA3789*, *CAG-95 sp009917455*, *Rikenella microfusus*, and *Parabacteroides_B_862066 goldsteinii*.

#### 2.8.4. The Impact of Inosine on Gut Microbiota Function

**Figure 11:**
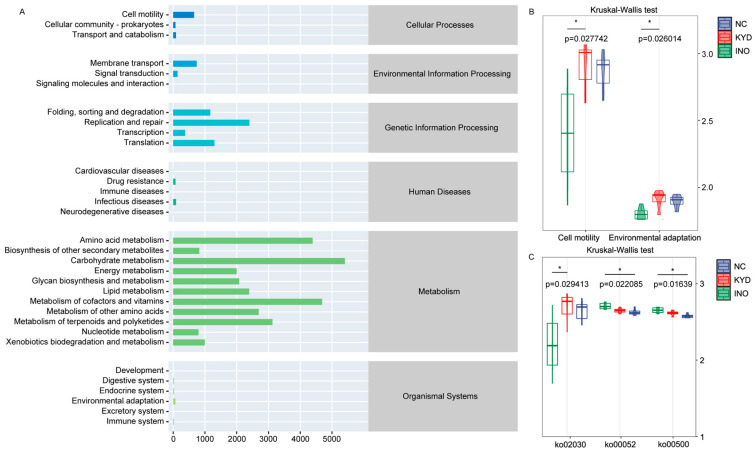
Microbial functional prediction (n = 6 per group). (**A**) Abundance statistics of metabolic pathways. (**B**) Differences in secondary metabolic pathways between groups. (**C**) Differences in tertiary metabolic pathways between groups. * *p* < 0.05.

The functional categories of colonic content can be broadly divided into six: Cellular Processes, Environmental Information Processing, Genetic Information Processing, Human Diseases, Metabolism, and Organismal Systems. Metabolism is the most represented category, followed by Genetic Information Processing, Environmental Information Processing, and Cellular Processes (Figure 11A). Differential analysis of secondary metabolic pathways within these categories, conducted via the KEGG database (Figure 11B), revealed a significant difference (*p* < 0.05) in Cell motility between the INO and KYD groups. This indicates that the functional capacity of the NC group was only slightly lower than that of the KYD group, a non-significant difference, whereas the INO group’s function was significantly lower than the KYD group’s. A similar trend was observed for Environmental adaptation, a secondary pathway within the Organismal Systems primary category, where the function of the NC group was marginally lower than the KYD group, and the INO group’s function was significantly lower, with a significant difference (*p* < 0.05) between the INO and KYD groups. Further analysis of tertiary metabolic pathways identified significant differences (*p* < 0.05) between the NC and INO groups for ko00052, associated with the branched-chain amino acid biosynthesis pathway, and ko00500, associated with the phenylalanine metabolism pathway. Additionally, ko02030, which is involved in amino acid transport, showed a significant difference (*p* < 0.05) between the KYD and INO groups.

### 2.9. Correlation Analysis of Gut Microbiota and Indicators

**Figure 12:**
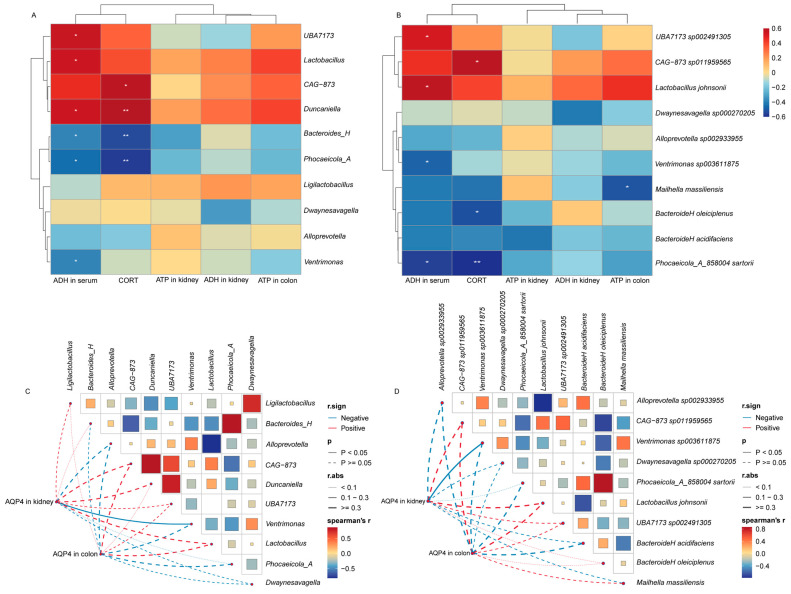
Correlation analysis. Correlation analysis of ADH, ATP, and CORT with (**A**) genus and (**B**) species (n = 6 per group). Correlation analysis of AQP4 levels with (**C**) genus and (**D**) species (n = 3 per group). * *p* < 0.05, ** *p* < 0.01.

As shown in Figure 12, a correlation analysis was performed between relevant indicators in the experiment and the top 10 most relatively abundant genera and phyla. The results (Figure 12A,B) showed that serum ADH levels were significantly negatively correlated with *Bacteroides_H*, *Phocaeicola_A*, and *Ventrimonas* (*p* < 0.05), while positively correlated with *Duncaniella*, *UBA7173*, and *Lactobacillus* (*p* < 0.05). Further correlation analysis with bacterial species revealed significant positive correlations with *UBA7173 sp002491305* and *Lactobacillus johnsonii* (*p* < 0.05), and significant negative correlations with *Phocaeicola_A_858004 sartorii* and *Ventrimonas sp0036111875* (*p* < 0.05). CORT was significantly and positively correlated with *CAG-873* and *Duncaniella* (*p* < 0.05), and significantly and negatively correlated with *Bacteroides_H* and *Phocaeicola_A* (*p* < 0.05). In correlation analysis with bacterial species, it was found to be significantly negatively correlated with *Phocaeicola_A_858004 sartorii* and *Bacteroides_H oleiciplenus* (*p* < 0.01, *p* < 0.05), and significantly positively correlated with *CAG-873 sp011959565* (*p* < 0.05). Meanwhile, colon ATP levels were also significantly negatively correlated with *Maihella massiiensis* (*p* < 0.05). The expression level of AQP4 showed significant negative correlations with *Ventrimonas* and *Ventrimonas sp003611874*, respectively (*p* < 0.05).

## 3. Discussion

### 3.1. Inosine Restores Metabolic Homeostasis and Modulates A2AR/AMPK/NF-κB-Associated Signaling in Kidney-Related Diarrhea

As an intermediate in purine metabolism, inosine facilitates ATP regeneration, supporting cellular energy demands under metabolic stress [23]. Aligned with this metabolic role, the mice with kidney-associated diarrhea exhibited reduced ATP levels, signifying compromised energy metabolism in this pathological state. However, inosine supplementation elevated tissue ATP concentrations, with a particularly notable increase in the colon. This suggests a partial improvement in cellular energy status following inosine treatment. Furthermore, inosine treatment led to significant increases in both CORT and ADH levels. Prior research has linked kidney-yang deficiency syndrome to dysfunction in the hypothalamic-pituitary-adrenal (HPA) axis and reduced ADH secretion [24,25]. CORT, the primary glucocorticoid controlled by the HPA axis, is crucial for metabolic adaptation, immune regulation, and stress responses [26,27]. Consequently, the restoration of CORT and ADH levels following inosine intervention likely reflects enhanced endocrine regulation and fluid balance. Collectively, the concurrent recovery of ATP, CORT, and ADH demonstrates that inosine treatment was associated with partial restoration of metabolic and physiological homeostasis. These results also suggest that inosine, acting as a purine metabolite involved in energy metabolism, may function to ameliorate symptoms in a mouse model of kidney-related diarrhea.

A previous study reveals that inosine confers neuroprotection by upregulating A2AR expression [28]. Activation of A2AR can alleviate renal inflammation and fibrosis [29], and can also improve gut inflammatory responses by modulation of ATP-mediated purinergic signaling pathways [30]. Furthermore, studies have also found that A2AR activation activates the AMPK/AKT pathway, reducing oxidative stress and improving cellular metabolic function [31]. Moreover, AMPK not only participates in ATP synthesis and energy metabolism regulation [32] but also maintains the integrity and normal function of the intestinal epithelial barrier [33]. In addition, activation of AMPK is accompanied by the inhibition of NF-κB signaling, effectively reducing the production of pro-inflammatory cytokines (such as IL-1β, IL-6, and TNF-α), and ultimately alleviating intestinal inflammation [34,35]. Consistent with these findings, in this experiment, after inosine intervention, the expression of A2AR and AMPK in the tissues of mice with renal-related diarrhea was increased, while the expression of NF-κB was decreased. These molecular changes were accompanied by elevated ATP levels, reduced inflammatory infiltration, and improved renal function. In summary, the process by which inosine restores metabolic homeostasis may be closely related to the activation of A2AR/AMPK signaling and the suppression of NF-κB-mediated inflammatory responses. Therefore, modulation of metabolic and inflammatory signaling may contribute to the beneficial effects of inosine in kidney-related diarrhea.

Furthermore, this study found that inosine intervention increased AQP4 levels. AQP4 is an important aquaporin protein that plays a key role in maintaining tissue water transport and fluid balance. Therefore, its abnormal expression is often associated with impaired water absorption and disrupted fluid homeostasis. Previous studies have shown that A2AR can regulate AQP4 expression, thereby affecting water transport processes [36]. Water transport mediated by AQPs is closely related to osmotic homeostasis regulated by ADH, and both are involved in maintaining the body’s fluid balance [37]. In this study, inosine intervention increased AQP4 expression, accompanied by elevated serum ADH levels. This suggests that inosine may contribute to the improvement of impaired fluid homeostasis during kidney-related diarrhea. However, the direct causal relationship among A2AR, AQP4, and ADH cannot be currently determined, and the related mechanisms still require further verification. Additionally, inosine can inhibit the release of pro-inflammatory mediators, thereby exerting anti-inflammatory effects [38]. Combined with the observed improvements in ATP, CORT, ADH, and AQP4 levels, as well as reduced tissue inflammation in this study, these findings suggest that inosine not only participates in energy metabolism regulation but may also promote homeostasis recovery by maintaining fluid balance and suppressing inflammatory responses.

In summary, the therapeutic effect of inosine on kidney-related diarrhea may be related to the coordinated regulation of metabolic homeostasis, fluid balance, and inflammatory responses, with A2AR/AMPK/NF-κB-related signaling potentially involved in this process.

### 3.2. Inosine Alters Gut Microbiota and Microbiota-Associated Metabolic Functions

Dysbiosis of the gut microbiota is typically characterized by a decrease in community richness and diversity, accompanied by a microbiota imbalance [39,40]. In a disease state, this is often closely associated with host metabolic dysfunction, enhanced inflammatory responses, and impaired intestinal barrier function [41]. This study found that compared to the NC group, the KYD group exhibited decreased Observed species, Chao1, Shannon, and Simpson indices, indicating a reduction in the richness and diversity of the gut microbiota. Concurrently, Rao’s quadratic entropy index increased, suggesting alterations in the gut microbiota’s structural composition [42]. Collectively, these findings demonstrate that renal-associated diarrhea is accompanied by an imbalance in the gut microecology. However, following inosine intervention, the gut microbiota did not fully return to the levels of the normal control group. Based on the results of α diversity, β diversity, and PCoA analysis, while the INO group developed a community distinct from the other two groups, its overall community structure remained highly similar to the KYD group. Nevertheless, the study observed significant improvements in the mice’s diarrhea symptoms, inflammatory status, and metabolic indicators. These findings suggest that improvements in diarrhea-associated symptoms, inflammatory status, and metabolic parameters do not necessarily coincide with the complete restoration of gut microbial composition. However, this observation does not demonstrate functional remodeling of the gut microbiota, as inosine may also directly affect host metabolic processes and inflammation-related signaling pathways. KEGG analysis revealed significant differences in the predicted abundances of pathways ko00052, ko00500, and ko02030, corresponding to galactose metabolism, starch and sucrose metabolism, and bacterial chemotaxis, respectively. The ko00052 and ko00500 may reflect differences in the ability of the microbiota to utilize carbohydrates, while changes in ko02030 may indicate alterations in the microbiota’s response to changes in the intestinal environmental signals. However, PICRUSt2 predicts microbial functional potential from 16S rRNA gene data and therefore cannot provide direct evidence of functional remodeling [43]. Further validation using metagenomic or metabolomic analyses is required. Accordingly, the changes in ATP, CORT, and ADH levels reflect secondary normalization rather than direct consequences of changes in microbial community function.

At the phylum level, inosine treatment significantly reduced the relative abundance of Firmicutes_A, while the relative abundance of Bacteroidota only showed an increasing trend. Previous studies have reported that both Firmicutes and Bacteroidota are involved in carbohydrate fermentation and contribute to the production of SCFAs [44]. Based on the group mean relative abundances, the Firmicutes/Bacteroidota ratio was elevated in the KYD group, whereas it showed a decreasing trend following inosine treatment. Although an increased Firmicutes/Bacteroidota ratio has been associated with inflammatory bowel disease in some studies [45], there is substantial functional heterogeneity within each phylum; thus, phylum-level abundance cannot directly reflect microbial metabolic activity or functional changes [46].

At the genus level, *Duncaniella* was significantly lower in the KYD group than in the NC group. Inosine treatment increased its relative abundance, although the difference between the KYD and INO groups was not statistically significant. Past research indicates that restored *Duncaniella* abundance often correlates with lower NF-κB activity and reduced inflammatory factors [47], a pattern also seen in hyperuricemia models [48]. Thus, *Duncaniella* may be associated with reduced inflammation; however, whether it contributes to the anti-inflammatory effects of inosine requires further investigation. Conversely, *Phocaeicola_A* and *Bacteroides_H* remained abundant in both the KYD and INO groups; this increase in these genera within the KYD group aligns with our prior experimental findings [16]. The same was true for the species *Phocaeicola_A_858004 sartorii* and *Bacteroides_H oleiciplenus*. Although these microbes may be tied to the disease, their persistence after inosine treatment suggests a complex, non-linear relationship between microbial composition and health improvement. Based on our KEGG findings, inosine likely works more by modulating microbial metabolic functions than by reversing the entire microbial makeup. LEfSe and random forest analyses identified characteristic microbes for each group: *Parabacteroides_B* and *Bacteroides_H* were prominent in the INO group, while *Paramuribaculum* and *Alistipes_A* defined the NC group. Studies show *Parabacteroides* are involved in breaking down carbohydrates and producing SCFAs [49], and *Bacteroides* are key to energy and nutrient use [50]. Therefore, inosine may be associated with differences in the microbial potential for nutrient utilization. Unfortunately, research on these specific bacterial species is still limited; they still require further validation through metagenomic or metabolomic analyses.

Correlation analysis revealed that multiple bacterial taxa were significantly associated with ADH levels. Among them, *Duncaniella*, *UBA7173*, and *Lactobacillus* showed a positive correlation with ADH, whereas *Bacteroides_H*, *Phocaeicola_A*, and *Ventrimonas* exhibited a negative correlation. Previous studies have indicated that *Lactobacillus* and its representative strain, *Lactobacillus johnsonii*, possess anti-inflammatory and intestinal protective effects [51,52,53], and are involved in the recovery from kidney injury [54]. Meanwhile, *Phocaeicola_A* has been associated with elevated expression of various inflammatory factors [55]. These results indicate that the gut microbiota is associated with host fluid homeostasis and inflammatory status. However, whether these microorganisms directly contribute to the regulation of these processes requires further investigation. In addition to ADH, this study also observed significant correlations between the gut microbiota and ATP, CORT, and AQP4. ATP is a key indicator of cellular energy metabolism, CORT reflects the endocrine regulatory state of the HPA axis, and AQP4 participates in the body’s water transport and maintenance of fluid balance. Therefore, these correlation findings further support a broad association between the gut microbiota and host metabolic homeostasis. Notably, *Maihella massiliensis* was negatively correlated with colonic ATP levels, while *Ventrimonas* and its related strains were negatively correlated with AQP4 expression. However, current functional research on these bacteria remains very limited; thus, their specific roles in host energy metabolism and fluid balance regulation require further validation.

In conclusion, our findings indicate that inosine treatment altered the composition of the gut microbiota and is associated with differences in functional potential. The current results do not confirm a causal relationship between changes in the microbial community and microbial metabolic pathways. Instead, the present findings suggest that inosine may exert direct effects on the host through modulation of the A2AR/AMPK/NF-κB signaling pathway, together with microbiota-associated alterations.

### 3.3. Limitations and Prospects

This study had a relatively limited sample size, and large biological differences existed among individuals, which may have reduced the statistical power of some indicators. Although some indicators showed clear trends of change, they did not reach statistical significance. Therefore, the stability and reproducibility of the results still need to be validated with a larger sample size in the future. In addition, this study used a model of diarrhea with kidney yang deficiency syndrome, rather than a model of organic kidney disease complicated by diarrhea, and its applicability to other types of kidney diseases still requires further verification. The functional prediction analysis in this study was inferred from 16S rRNA sequencing combined with the KEGG database; therefore, changes in related metabolic functions still need to be further verified by metabolomics or functional experiments. Furthermore, the changes in gut microbiota, improvements in metabolic indicators, and changes in A2AR/AMPK/NF-κB-related molecular expression observed in this study were mainly from correlation analysis, which is not sufficient to prove causality. Future studies should combine strategies such as fecal microbiota transplantation, metabolomics, metagenomics, and targeted microbiota intervention to further elucidate the interrelationships among inosine, gut microbiota, and microbiota-related metabolites and to clarify their roles in host energy metabolism, fluid balance, and inflammatory regulation. These studies will help provide a more solid basis for the application of inosine as a microbiota-related bioactive compound in nutritional interventions.

## 4. Materials and Methods

### 4.1. Drug and Kits Preparation

A kidney-related diarrhea model was established in male KM mice via oral co-administration of adenine (5 mg/mL, Batch No. EZ65564CEF, BioFRoxx, Einhausen, Germany) and *Folium sennae* (1 g/mL crude drug, Batch No. 2407032, Yulin, Guangxi, China). The adenine suspension was freshly prepared in sterile water. *Folium sennae* was decocted twice in sterile water, filtered, and combined. The combined decoctions were concentrated using the rotary evaporator (LC-RE-301, Shanghai Lichen Bangxi Instrument Technology Co., Ltd., Shanghai, China) at 75 °C to the final concentration, then stored at 4 °C [17]. Inosine (1 mg/mL, Sigma-Aldrich, St. Louis, MO, USA, HPLC ≥ 99%, Batch No. 0000317789) was freshly dissolved in 0.9% NaCl before use [22]. Serum and tissue levels of ADH, ATP, and CORT were measured using ELISA kits (Jiangsu Jingmei Biotechnology, Yancheng, Jiangsu, China, Cat. Nos. JM-02293M2, JM-11362M2, JM-02848M2). The main reagents used in this study included Hematoxylin and Eosin Staining Kit (Cat. No. BH0001), 4% paraformaldehyde (Cat. No. B0038), citrate antigen retrieval buffer (Cat. No. B0034), and DAB substrate kit (Cat. No. B0053), all purchased from Powerful Biology Co., Ltd. (Wuhan, China). TriQuick Reagent (Cat. No. R1100) and BSA (Cat. No. A8010) were obtained from Solarbio (Beijing, China). DEPC-treated water (Cat. No. R0022) was purchased from Beyotime Biotechnology (Shanghai, China). Evo M-MLV RT Mix Kit (Cat. No. AG11728) and SYBR Green Pro Taq HS premix qPCR kit (Cat. No. AG11739) were obtained from Accurate Biotechnology Co., Ltd. (Changsha, China). Hydrogen peroxide (Cat. No. 10011218), chloroform (Cat. No. 67-66-3), isopropanol (Cat. No. 80109218), and absolute ethanol (Cat. No. 10009218) were purchased from Sinopharm Chemical Reagent Co., Ltd. (Shanghai, China). The AQP4 antibody (rabbit, Cat. No. HA22672) was obtained from HuaBio (Hangzhou, China), and the HRP-conjugated goat anti-rabbit IgG (Cat. No. ab205718) was purchased from Abcam (Cambridge, UK). RIPA lysis buffer (Cat. No. BF0003), BCA Protein Assay Kit (Cat. No. BF0026), 5× reducing protein loading buffer (Cat. No. BF0007), SDS-PAGE Gel Preparation Kit (Cat. No. BF0006), TBST (Cat. No. B0065), and ECL reagent (Cat. No. BF0023) (Powerful Biology, Wuhan, China); 100 mM PMSF (Cat. No. BL507A) and non-fat milk (Cat. No. BS102) (Biosharp, Hefei, China); phosphatase inhibitor (Cat. No. MB12707, Meilunbio, Dalian, China); 0.45 μm PVDF membrane (Cat. No. W8040, BaiDaiBio, Changzhou, China); A2AR antibody (mouse, Cat. No. MT13299, Abmart, Shanghai, China); GAPDH antibody (rabbit, Cat. No. ET1601-4, HuaBio, Hangzhou, China); HRP-conjugated goat anti-mouse IgG (Cat. No. SA00001-1, Proteintech, Wuhan, China); and HRP-conjugated goat anti-rabbit IgG (Cat. No. 111-035-003, Jackson ImmunoResearch, West Grove, PA, USA).

### 4.2. Experimental Animals Grouping and Modeling

Thirty male KM mice (4 weeks old, 20 ± 2 g) were obtained from Hunan SJA Laboratory Animal Co., Ltd. and housed under standard specific pathogen-free conditions with five animals per cage and free access to food and water. After 3 days of acclimatization, mice were randomly assigned to three groups: normal control (NC, n = 10), kidney-related diarrhea model (KYD, n = 10), and inosine intervention (INO, n = 10). This experiment used the diarrhea with kidney-yang deficiency syndrome model established by our research group in previous studies [16]. Kidney-related diarrhea was induced by oral administration of adenine (50 mg/kg/day) for 14 days. From day 8, mice in the KYD and INO groups additionally received *Folium sennae* decoction (10 g/kg/day) by gavage for 7 days. The INO group was simultaneously treated with inosine (100 mg/kg/day) for 14 consecutive days starting on day 1 of model induction [22]. Mice in the NC and KYD groups received equivalent volumes of saline or sterile water.

Sample sizes varied across assays to optimize limited specimen utility. For each analysis, random animal selection from the original cohort minimized bias while enabling comprehensive parameter evaluation. Specific sample sizes (n) are detailed in respective figure legends.

### 4.3. General Observation and Measurement of Fecal Moisture Content

During the modeling period, mice were observed for mental state, activity, and stool traits. Their body weights were recorded daily, and rectal temperatures were measured. Fecal samples were collected individually from each mouse, weighed, dried at 110 °C for four hours, and reweighed. Fecal moisture content (%) = [(pre-drying wet weight − post-drying dry weight)/pre-drying wet weight] × 100 [56].

### 4.4. Measurement of BUN and Cr Levels in Serum

After the intervention, mice were anesthetized with isoflurane, and blood samples were collected from the retro-orbital sinus. The mice were subsequently euthanized by cervical dislocation. Blood samples were kept at room temperature for 4 h, centrifuged at 4 °C, 3000 rpm for 15 min, and the supernatants combined and stored. Prior to analysis, samples were thawed, re-centrifuged, and BUN and Cr levels were measured using an automated biochemical analyzer (Chemray 240, Rayto Life and Analytical Sciences Co., Ltd., Shenzhen, China) [55].

### 4.5. HE Staining for the Evaluation of Histopathological Alterations

Kidney and colon tissues were harvested, rinsed with saline, and fixed in 4% paraformaldehyde for 24 h. The entire kidney and proximal colon were harvested. The kidney was sectioned along its maximal longitudinal axis, whereas the colon was sectioned transversely. Following standard dehydration and paraffin embedding, the tissues were sliced into 4-µm sections with a rotary microtome (RM2016, Leica, Wetzlar, Germany). After deparaffinization and rehydration, the sections were stained with HE, then dehydrated, cleared, and mounted. Finally, the sections were observed and photographed using an optical microscope (Nikon Eclipse CI, Nikon, Tokyo, Japan) [57]. Histopathological alterations, including tissue architecture, epithelial integrity, and inflammatory cell infiltration, were qualitatively evaluated under light microscopy.

### 4.6. ELISA for Detection of ATP, ADH, and CORT Levels

A portion of colon and kidney tissue was harvested, washed with PBS solution chilled to 4 °C, and fragmented. Chilled PBS was added at the ratio specified by the ELISA kit, and the tissues were thoroughly homogenized with a homogenizer. The homogenized tissue was then centrifuged at 4 °C and 10,000 rpm for 3 min. The resulting supernatant was used for ELISA analysis [55]. Following the instructions for the ELISA kit, the levels of ADH and Cort in the processed serum, and of ADH and ATP in the kidney tissue and ATP in the colon tissue, were measured.

### 4.7. IHC Analysis of AQP4 Expression

The previously paraffin-embedded colon and kidney tissue sections were dewaxed and rehydrated. Antigen retrieval was performed using citrate antigen retrieval buffer. To block endogenous peroxidase activity, the sections were incubated with 3% hydrogen peroxide for 25 min, followed by blocking with 3% BSA at room temperature for 30 min. The sections were then incubated with an AQP4 primary antibody (1:1000) overnight at 4 °C in a humidified chamber. Subsequently, the sections were incubated with HRP-conjugated goat anti-rabbit IgG (1:2000) at room temperature for 50 min. Immunoreactivity was visualized using a DAB substrate kit, followed by counterstaining with hematoxylin. After dehydration, clearing, and mounting, the sections were examined under the optical microscope. For quantitative analysis, three non-overlapping fields per section were randomly selected and imaged at ×400 magnification under consistent imaging conditions. The average optical density (AOD) of AQP4-positive staining was quantified using ImageJ 1.54p (National Institutes of Health, Bethesda, MD, USA), and the resulting mean AOD values were used for statistical analysis [16].

### 4.8. RT-qPCR Detection of AMPK and NF-κB mRNA in Colon and Kidney

Approximately 30–50 mg of colon or kidney tissue was homogenized in TriQuick Reagent and lysed. After phase separation with chloroform, the aqueous phase was collected, and total RNA was precipitated with isopropanol, washed with 75% ethanol, dissolved in DEPC-treated water, and stored at −80 °C. Reverse transcription was performed using the Evo M-MLV RT Mix Kit according to the instructions in a total reaction volume of 20 μL. The reaction conditions were as follows: 37 °C for 15 min, 85 °C for 5 s, and a hold at 4 °C. Quantitative real-time PCR was performed using SYBR Green Pro Taq HS premix qPCR kit on the Real-Time Quantitative PCR System (Quant Gene 9600, BIOER, Hangzhou, China). Each reaction was performed in a total volume of 20 μL containing 10 μL of 2× SYBR Green Premix, 2 μL of primer mixture, 2 μL of cDNA template, and 6 μL of nuclease-free water. The amplification program consisted of an initial denaturation at 95 °C for 30 s, followed by 40 cycles of 95 °C for 5 s and 60 °C for 30 s. A melting curve analysis was performed from 60 °C to 95 °C. Relative mRNA expression levels were calculated using the 2^−ΔΔCt method with GAPDH as the internal reference gene [58]. All reactions were performed in triplicate. Primer sequences are listed in Table 1.

### 4.9. WB Measures A2AR Expression Levels in the Kidney and Colon

Tissues were homogenized in lysis buffer supplemented with PMSF and phosphatase inhibitor, subjected to three rounds of grinding and vortexing, centrifuged, and the supernatant collected. Protein concentrations were determined using a BCA Protein Assay Kit. Protein samples were mixed with 5× reducing protein loading buffer, denatured by boiling, and stored at −80 °C. SDS-PAGE gels were prepared using an SDS-PAGE Gel Preparation Kit. SDS-PAGE gels were prepared, loaded with samples, and run at 75 V and 100 V, respectively. The membranes were blocked with 5% non-fat milk and incubated overnight at 4 °C with primary antibodies against A2AR (1:500) and GAPDH (1:5000). After washing with TBST, the membranes were incubated for 30 min at room temperature with the corresponding HRP-conjugated secondary antibodies: HRP-goat anti-mouse IgG (1:5000) for A2AR and HRP-goat anti-rabbit IgG (1:5000) for GAPDH. Protein bands were visualized using ECL reagent with a Tanon 4800 chemiluminescence imaging system (Tanon Science & Technology, Shanghai, China) [59]. Band intensities were quantified using ImageJ and normalized to determine relative protein expression levels.

### 4.10. 16S rRNA Sequencing of Colon Content Microbiota

Mouse colonic contents were aseptically harvested and processed with extraction lysis solution. Nucleic acids were isolated using the MagBeads FastDNA Kit (116564384) (MP Biomedicals, Irvine, CA, USA), with DNA quantified and sized via electrophoresis and Nanodrop. The V3-V4 region of bacterial 16S rRNA was amplified with primers 338F (5′-ACTCCTACGGGAGGCAGCA-3′) and 806R (5′-GGACTACHVGGGTWTCTAAT-3′) using NEB Q5 polymerase (25 cycles: 98 °C/30s, 52 °C/30s, 72 °C/45s, final extension 72 °C/5min). PCR products were verified, excised, quantified with PicoGreen, and pooled. Libraries were constructed using Illumina TruSeq Nano kit (Cat. No. 20015965; Illumina, San Diego, CA, USA), quantified with Qubit, sized with Agilent 2100, normalized to 10 nM, and sequenced on Illumina NovaSeq (2 × 250 bp). Bioinformatics employed QIIME2 2024.5 (QIIME 2 Development Team, Seattle, WA, USA) with cutadapt and DADA2 for demultiplexing, quality filtering, denoising, and ASV generation [60]. Sequencing was performed by Shanghai Personal Biotechnology Co., Ltd. (Shanghai, China).

Microbiota Composition Analysis: Using the Greengenes2 database, ASV feature sequences were compared with reference sequences to obtain taxonomic information and remove rare ASVs. Taxonomic composition at each level was obtained based on ASV division and identification. R 4.4.3 (R Foundation for Statistical Computing, Vienna, Austria) was used to create bar charts comparing microbial groups across samples. QIIME2 generated composition and abundance tables, with bar charts showing inter-group differences. LEfSe detected differentially abundant taxonomic units. Microbial community abundance differential analysis, LEfSe analysis, functional prediction, and random forest analysis were conducted using R 4.4.3.

Alpha diversity analysis: Alpha diversity was assessed using QIIME2 based on the rarefied ASV abundance table. To minimize bias caused by differences in sequencing depth, the ASV table was rarefied to 95% of the sequence count of the sample with the lowest sequencing depth. Data visualization was performed using R (version 4.3.3). Differences in alpha diversity among groups were evaluated using the Kruskal–Wallis test, followed by Dunn’s post hoc test for pairwise comparisons.

Beta diversity analysis: Beta diversity was calculated using QIIME2 based on the ASV abundance table. A Jaccard distance matrix was constructed, and PCoA was performed to visualize differences in microbial community composition among groups.

Metabolic function prediction: Functional profiles of the gut microbiota were predicted using PICRUSt2 based on the ASV abundance table and annotated against the KEGG database. Differences in KEGG pathway abundance among groups were analyzed using the Kruskal–Wallis test, followed by BH correction for multiple testing and Dunn’s post hoc test for pairwise comparisons.

### 4.11. Statistical Analysis

Data analysis was conducted using SPSS 25.0 (IBM Corp., Armonk, NY, USA) and GraphPad Prism 10.1.2 (GraphPad Software, San Diego, CA, USA). Initially, the data were subjected to normality and homogeneity of variance tests. Data meeting these assumptions were presented as mean ± standard deviation. For data satisfying normality and homogeneity of variance, one-way analysis of variance (ANOVA) was employed, followed by Tukey’s multiple comparison test for pairwise comparisons; otherwise, the Kruskal–Wallis test was applied. Differential abundance analyses of the gut microbiota and predicted functional pathways (KEGG and MetaCyc) were performed using the Kruskal–Wallis test, combined with Dunn’s multiple comparison test. Correlations between the gut microbiota and physiological or biochemical parameters were assessed via Spearman’s rank correlation analysis. A *p*-value < 0.05 was considered statistically significant. Figures were generated using GraphPad Prism 10.1.2 and Adobe Illustrator 2024 (Adobe Inc., San Jose, CA, USA). Significance levels: * *p* < 0.05, ** *p* < 0.01, *** *p* < 0.001.

## 5. Conclusions

This study showed that inosine alleviated pathological features of kidney-related diarrhea in mice. After treatment with inosine, levels of ATP, CORT, and ADH were restored. This was associated with increased expression of A2AR and AMPK and decreased expression of NF-κB, suggesting that inosine may exert protective effects by regulating metabolic homeostasis and inflammatory responses. Additionally, inosine enhanced the expression of AQP4 in colonic and renal tissues, implying its role in maintaining fluid balance and improving water transport function. Moreover, inosine changes the gut microbiota composition and its predicted metabolic pathways. Correlation analysis indicated that these microbial variations are linked to host metabolism, inflammation, and fluid balance metrics. In summary, inosine likely alleviates kidney-related diarrhea through a combination of mechanisms: modulating the gut microbiota, improving metabolic homeostasis, and influencing the A2AR/AMPK/NF-κB signaling pathways. These findings provide preliminary evidence supporting further investigation of inosine as a potential bioactive intervention for kidney-related diarrhea.

## Figures and Tables

**Table 1:** Primer sequences used for RT-qPCR analysis.

Primer	Primer F	Primer R	Product Length (bp)
GAPDH	TGCCCCCATGTTTGTGATG	TGTGGTCATGAGCCCTTCC	151
AMPK	AAGTGAAGGTGGGCAAGCAC	GGCTTTCCTTTTCGTCCAACC	264
NF-κB	ACCTGTTCCAAAGAGCACCC	CAAGGCCCCCAAGTCTTCAT	94

## Data Availability

The original sequence data were submitted to the Sequence Read Archive of the NCBI database, NO. PRJNA1434102 (https://www.ncbi.nlm.nih.gov, accessed on 9 March 2026).

## Abbreviations

The following abbreviations are used in this manuscript:

IMP	inosine monophosphate
AMP	Adenosine monophosphate
ATP	Adenosine triphosphate
AMPK	AMP-activated protein kinase
A2AR	Adenosine A2A Receptor
CORT	Corticosterone
ADH	Antidiuretic hormone
AQP4	Aquaporin-4
IHC	Immunohistochemistry
BUN	blood urea nitrogen
Cr	creatinine
HE	Hematoxylin and eosin
ASVs	Amplicon Sequence Variants
LEfSe	Linear Discriminant Analysis Effect Size
PCoA	Principal Coordinate Analysis
WB	Western Blot
HPA	hypothalamic–pituitary–adrenal
SCFAs	short-chain fatty acids
